# Clinical, metabolomic, and proteomic profiles associated with reproductive outcomes in unexplained recurrent pregnancy loss

**DOI:** 10.3389/fendo.2026.1875194

**Published:** 2026-07-10

**Authors:** Zicheng Song, Yishi Jiang, Zhixing Zhu, Yan Che, Shuping Li, Aimin Zhao

**Affiliations:** 1 Department of Obstetrics and Gynecology, Renji Hospital, Shanghai Jiao Tong University School of Medicine, Shanghai, China; 2 Shanghai-MOST Key Laboratory of Health and Disease Genomics, NHC Key Laboratory of Reproduction Regulation, Shanghai Institute for Biomedical and Pharmaceutical Technologies, Shanghai, China; 3 Shanghai Engineering Research Center for Big Data in Pediatric Precision Medicine; Center for Biomedical Informatics, Shanghai Children’s Hospital; School of Medicine, Shanghai Jiao Tong University, Shanghai, China; 4 Department of Obstetrics and Gynecology, Changzhou Hospital Affiliated to Nanjing University of Chinese Medicine, Changzhou, China

**Keywords:** bile acids, caffeine, metabolomics, prolactin, proteomics, reproductive outcome, testosterone, unexplained recurrent pregnancy loss

## Abstract

**Objective:**

To identify preconception clinical and multi-omics factors associated with conception and early pregnancy loss in women with unexplained recurrent pregnancy loss (URPL).

**Methods:**

In this prospective cohort study, 149 women with URPL selected from 420 outpatients based on guideline-recommended criteria were enrolled between November 2024 and May 2025 and followed-up for 12 months. Preconception fasting plasma was analyzed for clinical biomarkers, untargeted metabolomics, and data-independent acquisition proteomics. Outcomes included conception, ongoing pregnancy beyond 12 weeks and early pregnancy loss before 12 weeks. Multivariable logistic regression was used to evaluate the associations of clinical and multi-omics factors with reproductive outcomes, adjusting for maternal age, body mass index, number of prior losses, and use of assisted reproductive technology.

**Results:**

Of 149 women, 99 conceived (66.4%) during the follow-up. By 12 weeks of gestation, 67 (67.7%) had ongoing pregnancies and 32 (32.3%) experienced early pregnancy loss. Higher testosterone was associated with lower probability of conception (adjusted odds ratio [aOR] 0.50, 95% confidence interval [CI] 0.28–0.89, *p* = 0.019). Women who conceived showed higher levels of progesterone-related metabolites, including 17-hydroxyprogesterone (fold change, FC = 3.89), pregnanediol 3-O-glucuronide (FC = 2.37), and pregnanetriol 3α-O-β-D-glucuronide (FC = 2.39). Among women who conceived, higher prolactin was associated with higher odds of early pregnancy loss (aOR 1.09, 95% CI 1.01–1.18, *p* = 0.036), and anti-phosphatidylserine/prothrombin showed a borderline association (aOR 1.07, 95% CI 1.00–1.14, *p* = 0.052). Early pregnancy loss was characterized by lower bile acid-related metabolites and higher caffeine and methylxanthine metabolites. Proteomic analysis showed enrichment of bile acid biosynthetic process. After adjustment, higher 7α,12α-dihydroxy-3-oxocholest-4-en-27-oic acid was associated with lower odds of early pregnancy loss (aOR 0.64, 95% CI 0.44–0.95, *p* = 0.025).

**Conclusions:**

Higher testosterone was associated with lower odds of conception. Among women who conceived, early pregnancy loss was associated with higher prolactin, borderline higher anti-PS/PT, lower bile acid-related metabolites, and higher caffeine-related metabolites, with 7α,12α-dihydroxy-3-oxocholest-4-en-27-oic acid identified as an exploratory metabolomic candidate. These findings suggest that potential androgen-related biology, prolactin, non-criteria antiphospholipid antibodies, and bile acid metabolism may be associated with reproductive outcomes in URPL, warranting validation in independent cohorts.

## Introduction

Recurrent pregnancy loss (RPL) is a distressing reproductive disorder that poses a substantial physical and psychological burden (1, 2). Although definitions vary internationally, most guidelines define RPL as two or more consecutive pregnancy losses, whereas others adopt a threshold of three losses while still allowing earlier evaluation in high-risk populations (3–5). Established causes of RPL are routinely evaluated in the diagnostic work-up. Parental chromosomal rearrangements and embryonic aneuploidy are well-recognized genetic contributors; uterine anatomical anomalies, such as a septate uterus, can impair implantation and placentation; and endocrine disorders, including overt thyroid dysfunction, poorly controlled diabetes, and hyperprolactinemia, can disturb the hormonal environment required for implantation and luteal support (1, 3, 4). Antiphospholipid syndrome (APS), defined by persistent positivity for antiphospholipid antibodies, including lupus anticoagulant, anticardiolipin, and anti-β2-glycoprotein I antibodies, is the most established immune-thrombotic cause of recurrent pregnancy loss and may contribute to injury at the maternal–fetal interface (6).

Despite comprehensive evaluations recommended by current guidelines, approximately half of affected couples remain without an identifiable etiology, a condition classified as unexplained RPL (URPL) (7). The roles of several additional candidate factors, including prolactin (PRL) and non-criteria antiphospholipid antibodies such as anti-phosphatidylserine/prothrombin (anti-PS/PT), have been suggested but not firmly established, and guidance on their routine testing remains inconsistent across societies (4, 8, 9).

The clinical management of URPL remains challenging. Prognosis is highly heterogeneous at the individual level (10). In clinical practice, delayed conception adds another layer of complexity beyond early pregnancy maintenance. Current preconception counseling relies primarily on maternal age and pregnancy loss history, offering limited prognostic discrimination. This uncertainty often drives empiric therapeutic interventions despite limited supporting evidence (11, 12). Moreover, although endocrine, metabolic, and immunological factors are frequently investigated in research settings, the ESHRE guideline advises against several commonly used tests because of their limited prognostic value.

The limited predictive value of conventional testing is likely to reflect the biological complexity of early pregnancy maintenance (13). Emerging evidence suggests that URPL is a highly heterogeneous condition. Its pathogenesis may involve isolated abnormalities or combinations of immunological, endometrial, placental, metabolic, and coagulative dysfunctions, which cannot be adequately captured by single-focus clinical studies (1). As a result, isolated biomarkers often show inconsistent associations and fail to capture the coordinated biological processes that influence reproductive outcomes. Multi-omics profiling provides an opportunity to characterize this complexity more systematically by integrating multiple layers of biological information (14).

Importantly, prognostic evaluation in URPL has been hampered by a reliance on retrospective study designs, which may conflate cause with consequence (14, 15). To identify genuine preconception factors, patient profiles need to be assessed before conception rather than after the miscarriage (16). Accordingly, there is a clear need for prospective, outcome-anchored studies evaluating both baseline clinical characteristics and multi-omics profiles.

To address these gaps, we established a prospective URPL cohort, excluding recognized etiologies using standardized criteria. By integrating baseline clinical and multi-omics characteristics, our primary objective was to identify candidate factors associated with reproductive outcomes in URPL, including both conception success and early pregnancy maintenance.

## Materials and methods

### Study design and participants

This prospective cohort study was conducted at the Reproductive Immunology Clinic of Renji Hospital, Shanghai Jiao Tong University School of Medicine. Women aged 18 to 45 years with a history of at least two consecutive spontaneous pregnancy losses before 12 gestational weeks with the same partner were enrolled between November 2024 and May 2025.

URPL was confirmed after completion of guideline-recommended evaluation and strict exclusion of established etiologies. Specifically, the exclusion criteria included: (1) chromosomal abnormalities in either partner; (2) uncorrected uterine anatomical malformations; (3) endocrine disorders such as uncontrolled thyroid disease, diabetes mellitus, or uncontrolled hyperprolactinemia; (4) recognized autoimmune diseases or thrombophilia, including antiphospholipid syndrome (APS), confirmed by positive antiphospholipid antibodies (anticardiolipin, anti-β2-glycoprotein I, lupus anticoagulant) on two or more occasions at least 12 weeks apart according to the 2023 ACR/EULAR classification criteria, or systemic lupus erythematosus. Patients who tested positive for a single standard antiphospholipid antibody (aPL) at one time point but reverted to negative on confirmatory retesting (≥12 weeks later) without intervention were not classified as APS and remained eligible for enrollment; (5) active infections; and (6) severe systemic conditions such as uncontrolled hypertension, renal or hepatic dysfunction, or severe psychiatric disorders.

### Clinical data and biospecimen collection

Baseline demographic and clinical characteristics were obtained at enrollment. Peripheral fasting blood samples were collected from all participants in the non-pregnant state, before initiation of any therapeutic interventions. Routine laboratory assessments included sex hormones such as testosterone, prolactin, fasting glucose, renal function estimated by glomerular filtration rate (eGFR), homocysteine, platelet aggregation, and thyroid function including thyroid-stimulating hormone (TSH) and thyroid peroxidase antibody (TPO-Ab). In addition, a comprehensive autoantibody panel was assessed, including standard antiphospholipid and anti-PS/PT antibodies. Anti-PS/PT antibodies (IgM/IgG) were tested by semiquantitative QUANTA Lite^®^ ELISA kits provided by Inova Diagnostics, Inc (San Diego, CA, USA) (17).

For omics analysis, plasma was isolated at 1600 g for 10 min at 4 °C within 2 hours of collection, aliquoted, and stored immediately at −80 °C until subsequent untargeted metabolomic and data-independent acquisition (DIA) proteomic analyses were performed.

### Follow-up procedures and endpoint definitions

Participants were followed for 12 months through routine clinic visits and regular follow-up contacts. Clinical management during follow-up was provided as part of routine care at the treating physician’s discretion. Pharmacological interventions and use of assisted reproductive technology (ART, including IVF/ICSI/PGT-A) were recorded for covariate adjustment.

We prespecified two outcomes. The first endpoint was conception during follow-up, defined by a positive serum β−hCG test. The second endpoint, evaluated among women who conceived, was the early pregnancy outcome of the index pregnancy, classified as either ongoing pregnancy or early pregnancy loss. Ongoing pregnancy was defined as ultrasound-confirmed fetal cardiac activity persisting beyond 12 gestational weeks. Early pregnancy loss was defined as loss of an intrauterine pregnancy before 12 weeks, including biochemical pregnancy loss and clinical miscarriage. Cases involving ectopic pregnancy or recurrent implantation failure were excluded from the outcome analyses.

For multi-omics analyses and visualization, participants were divided into three groups based on reproductive outcomes: Group A (ongoing pregnancy beyond 12 weeks), Group B (early pregnancy loss before 12 weeks), and Group C (non-conception during follow-up).

### Untargeted metabolomics profiling

Analyses were performed on an ACQUITY UPLC I−Class system (Waters Corporation, Milford, USA) coupled to a high-resolution mass spectrometer (Thermo Fisher Scientific, Waltham, MA, USA). Chromatographic separation was performed on an ACQUITY UPLC HSS T3 column (100 mm × 2.1 mm, 1.8 µm; Waters, Milford, MA, USA) in both positive and negative ion modes. The mobile phases consisted of 0.1% formic acid in water (A) and acetonitrile (B), delivered at a flow rate of 0.35 mL/min. The solvent gradient was as follows: 0–2.0 min, 5% B; 2.0–4.0 min, 5%–30% B; 4.0–8.0 min, 30%–50% B; 8.0–10.0 min, 50%–80% B; 10.0–14.0 min, 80%–100% B; 14.0–15.0 min, 100% B; 15.0–15.1 min, 100%–5% B; and 15.1–16.0 min, 5% B for equilibration.

The mass spectrometer operated in heated electrospray ionization mode with a spray voltage of 3.8 kV (positive)/3.2 kV (negative) and a capillary temperature of 320 °C. To ensure analytical robustness, quality control (QC) samples (pooled aliquots of all study samples) were injected periodically. Raw data were processed for peak alignment and integration. A three-step filtering pipeline was applied: (1) Stability Filter: Ion peaks with a relative standard deviation (RSD) >30% in QC samples were removed; (2) Missing Value Filter: Metabolites with > 50% missing values in any group were excluded; and (3) Imputation and Normalization: Remaining missing values, recorded as zeros, were imputed using half the minimum observed value for that feature. The final data matrix was log2-transformed before statistical analysis.

### Data-independent acquisition proteomics

Plasma proteins were profiled using a label-free DIA strategy. High-abundance proteins were depleted, and samples were digested with trypsin. Peptides were analyzed on a Thermo Scientific Vanquish Neo UHPLC system coupled to an Orbitrap Astral mass spectrometer (Thermo Fisher Scientific, Waltham, MA, USA). Data were acquired in DIA mode to maximize proteome coverage. Raw data were processed using DIA−NN (version 2.1) against a human reference database. Proteins with > 50% missing values in any group were excluded. Remaining missing values were not imputed at the filtering stage to preserve data integrity for subsequent differential analysis.

### Multi-omics analyses

To further characterize biological features associated with reproductive outcomes, we performed orthogonal partial least squares discriminant analysis (OPLS-DA) and assessed variable importance in projection to rank discriminatory features. Univariate P values were adjusted using the Benjamini-Hochberg False Discovery Rate (FDR) method. Candidate metabolites for exploratory interpretation were selected using VIP > 1.5, fold change (FC) ≥ 1.5 or FC ≤ 0.67, and *nominal p* < 0.05; FDR-adjusted q-values were reported to contextualize multiplicity. Differentially expressed proteins (DEPs) were defined by FC ≥ 2 or FC ≤ 0.5 and *nominal p* < 0.05, with q-values reported for reference. Correlations between candidate metabolites and prespecified clinical indicators were assessed using Pearson correlation coefficients, with FDR adjustment across tested correlations. Given the modest sample size relative to the number of features tested, individual omics findings were interpreted as candidate signals, and proteomic results were interpreted primarily at the pathway-enrichment level through functional enrichment and biologically annotated protein groups rather than as individually validated biomarkers. Functional enrichment for proteomics was performed using Gene Ontology (GO). Metabolite pathway enrichment and joint pathway analysis across metabolomics and proteomics were based on KEGG.

### Statistical analysis

Baseline continuous clinical characteristics were presented as means ± standard deviations (SD) or medians with interquartile ranges (IQR), depending on distribution. Categorical variables were presented as frequency and percentage. Missing values in clinical covariates were handled using multiple imputations by chained equations (MICE). Continuous variables were compared using analysis of variance (ANOVA) or the Kruskal–Wallis test, and categorical variables using chi-square or Fisher’s exact test. Associations between biomarkers and early pregnancy loss were assessed using logistic regression. Variables *with p* < 0.1 in the univariate analysis were included in multivariable models, adjusted for maternal age, body mass index (BMI), number of prior pregnancy losses, and ART use. Factors retained from the clinical and metabolomic models were subsequently entered into a final exploratory combined multivariable model.

Given the limited number of early pregnancy loss events, the integrated clinical–metabolomic is performed as explicitly exploratory and should not be interpreted as a validated prediction model, risk score, or clinical decision-making tool. Results are reported as odds ratios (ORs) with 95% confidence intervals (CIs). For multi-omics analyses, statistical significance was determined using FDR-adjusted *p*-values as described above. For clinical logistic regression models, *p* < 0.05 was considered statistically significant. All analyses were performed using R software (version 4.2.1).

### Ethics approval

The study protocol was approved by the Ethics Committee of Renji Hospital (LY2024−258−B) and conducted in accordance with the Declaration of Helsinki. Written informed consent was obtained from all participants prior to enrollment.

## Results

### Study population and pregnancy outcomes

**Figure 1:**
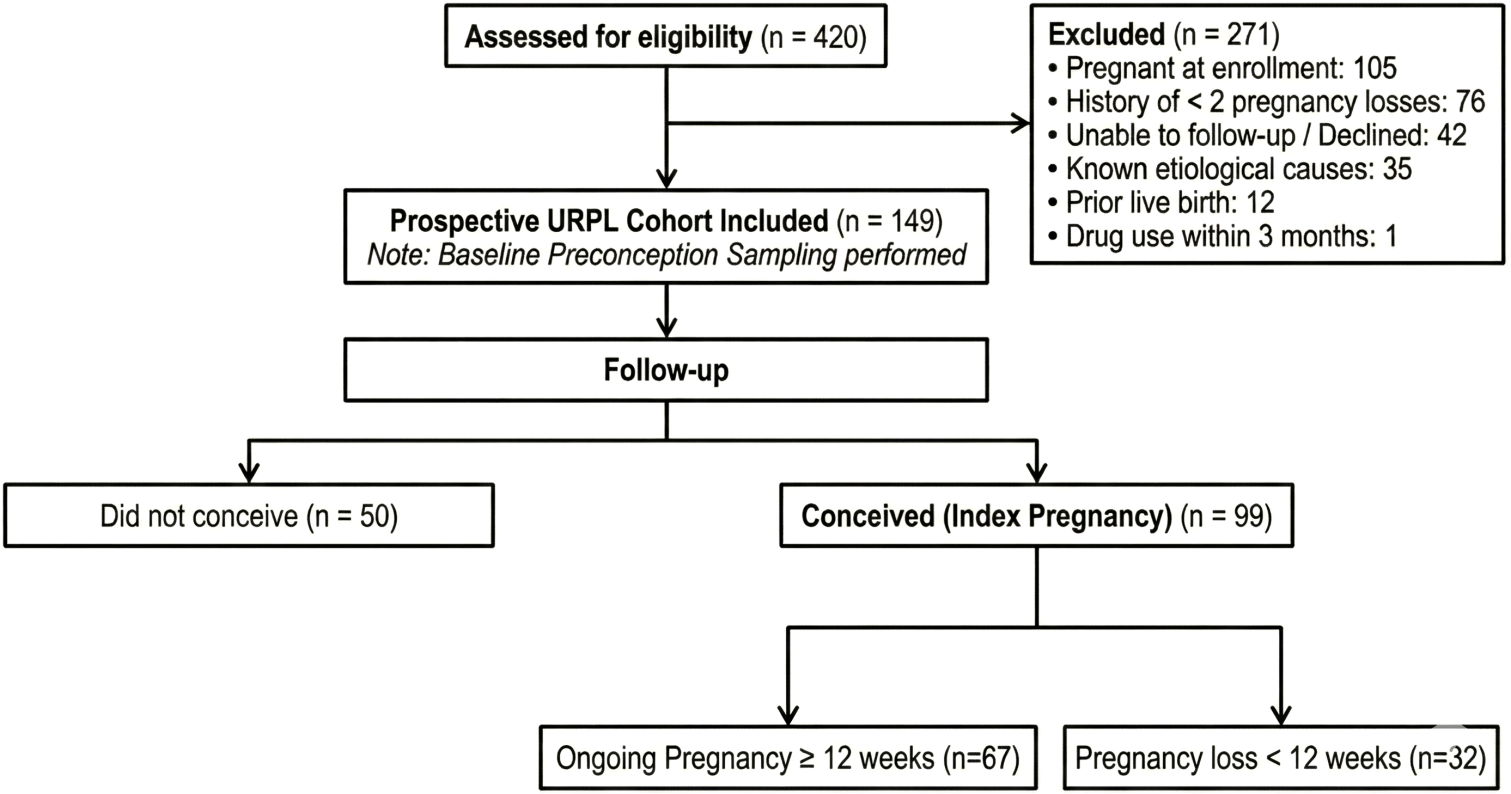
Study flowchart. A schematic diagram illustrating the participant selection process, follow-up, and pregnancy outcomes for the prospective cohort of women with URPL. A flowchart showing 420 women screened, 271 excluded according to predefined criteria, 149 enrolled with multi-omics sampling, 99 conceiving during 12-month follow-up, of whom 67 had ongoing pregnancies beyond 12 weeks and 32 had early pregnancy losses; 50 women did not conceive during follow-up. URPL, unexplained recurrent pregnancy loss.

A total of 420 women with recurrent pregnancy loss were screened for eligibility. After excluding 271 patients according to the predefined criteria, 149 women with URPL (35.5%) were prospectively enrolled and completed baseline multi-omics sampling (**Figure 1**). During the 12-month follow-up period, 99 participants (66.4%) conceived. The conception group (Group A+B) comprised 67 women (67.7%) with ongoing pregnancy beyond 12 gestational weeks (Group A) and 32 women (32.3%) with early pregnancy loss before 12 gestational weeks (Group B). The remaining 50 women did not conceive during follow-up (Group C). Consequently, analyses of conception compared women who conceived (Groups A+B) with women who did not conceive (Group C), whereas analyses of early pregnancy loss were confined to Group B with Group A. The median time from enrollment to conception was 3.0 months (IQR, 1.3–5.5).

### Baseline characteristics and clinical management

Baseline demographic and clinical characteristics, stratified by reproductive outcome, are shown in **Table 1**. The three groups were generally comparable with respect to maternal age *(p =* 0.260), BMI *(p =* 0.627), and the number of prior losses *(p =* 0.600). Significant differences across groups were observed in serum testosterone levels *(p =* 0.019) and ADP-induced platelet aggregation rates *(p =* 0.047). Specifically, the non-conception group exhibited the highest median testosterone level, although values in all groups remained within the laboratory reference range. A borderline difference was observed for fasting glucose *(p =* 0.057). Anti-PS/PT IgM levels were numerically lower in the ongoing pregnancy group compared to the early pregnancy loss group, although the overall between-group difference did not reach statistical significance *(p =* 0.248). Other clinical characteristics were comparable across groups (all *p* > 0.1).

**Table 1:** Baseline characteristics stratified by reproductive outcome.

Variables	Ongoing pregnancy (Group A, n=67)	Pregnancy loss (Group B, n=32)	Non-conception (Group C, n=50)	*p*
Demographics				
Age ( $\bar{x}$ ± SD)	31.66 ± 3.62	32.88 ± 3.10	32.14 ± 3.44	0.260
BMI, ( $\bar{x}$ ± SD)	22.03 ± 2.57	21.65 ± 2.85	21.57 ± 2.82	0.627
ART use, n (%)	13 (19.4)	10 (31.2)	10 (20.0)	0.375
No. of previous losses, median (IQR)	2.00 (2.00-3.00)	2.00 (2.00-3.00)	2.00 (2.00-3.00)	0.600
Gestational age of prior losses (weeks), median (IQR)	6.50 (5.67-7.50)	6.50 (5.88-8.05)	6.67 (5.69-8.00)	0.777
Baseline biomarkers				
Anti-PS/PT IgG (units), median (IQR)	6.45 (5.35-9.34)	6.97 (5.80-8.70)	7.39 (6.20-8.49)	0.375
Anti-PS/PT IgM (units), median (IQR)	18.01 (12.61-23.91)	20.63 (14.84-28.80)	18.47 (14.20-23.96)	0.248
TSH (mU/L), median (IQR)	1.98 (1.36-2.78)	1.86 (1.37-2.52)	1.90 (1.36-2.54)	0.885
Testosterone (nmol/L), median (IQR)	1.42 (1.04-2.01)	1.27 (0.97-1.62)	1.60 (1.27-2.10)	0.019*
Prolactin (ng/mL), median (IQR)	14.21 (11.41-19.68)	16.67 (13.63-22.62)	15.56 (12.41-20.02)	0.175
Fasting Glucose (mmol/L), median (IQR)	4.78 (4.52-5.04)	5.01 (4.71-5.15)	4.89 (4.69-5.10)	0.057
Platelet Aggregation (AA) (%), median (IQR)	84.30 (80.85-88.00)	82.55 (73.45-85.32)	82.20 (77.30-86.10)	0.147
Platelet Aggregation (ADP) (%), median (IQR)	82.30 (76.30-85.50)	78.70 (71.15-81.70)	80.70 (75.77-83.75)	0.047*
eGFR (mL/min/1.73m²), median (IQR)	122.00 (119.00-125.00)	120.00 (115.00-124.00)	121.00 (118.00-124.00)	0.133
Homocysteine (μmol/L), median (IQR)	7.40 (6.35-8.45)	7.90 (7.00-9.20)	7.70 (6.70-10.00)	0.228
TPO-Ab Positive, n (%)	6 (9.1)	3 (10.3)	2 (4.5)	0.607
Therapeutic regimen				0.028*
No Intervention, n (%)	11 (16.4)	11 (34.4)	22 (44.0)	
Anticoagulant Monotherapy, n (%)	5 (7.5)	3 (9.4)	1 (2.0)	
Immunomodulatory Monotherapy, n (%)	26 (38.8)	11 (34.4)	17 (34.0)	
Combination Therapy, n (%)	25 (37.3)	7 (21.9)	10 (20.0)	

Data are presented as mean (SD), median (IQR), or n (%). *The p*-values were calculated using ANOVA or Kruskal-Wallis tests for continuous variables, and chi-square or Fisher’s exact tests for categorical variables. BMI, body mass index; ART, assisted reproductive technology; anti-PS/PT, anti-phosphatidylserine/prothrombin; TSH, thyroid-stimulating hormone; TPO-Ab, thyroid peroxidase antibody; eGFR, estimated glomerular filtration rate; AA, arachidonic acid; ADP, adenosine diphosphate. TPO-Ab positivity was defined as TPO-Ab above 34 IU/mL. *p < 0.05.

Use of ART did not differ significantly among the outcome groups *(p =* 0.375). Pharmacological interventions differed across the three groups *(p =* 0.028), largely because a higher proportion of patients in the non-conception group received no treatment. However, among women who conceived, the use of anticoagulant, immunomodulatory, or combined therapies was comparable between the ongoing pregnancy and early pregnancy loss groups (*all p* > 0.05).

### Clinical factors associated with conception

Logistic regression identified baseline testosterone levels as inversely associated with conception (OR 0.51, 95% CI 0.29-0.86, *p* = 0.014) (**Table 2**). After adjustment for maternal age, BMI, number of pregnancy losses, and ART use, testosterone remained the only variable associated with conception in the adjusted model (adjusted OR 0.50, 95% CI: 0.28–0.89, *p* = 0.019). Other demographic characteristics and clinical biomarkers were not significantly associated with higher odds of conception (*all p* > 0.1). These results suggest that the androgenic profile warrants further evaluation in the URPL population.

**Table 2:** Multivariable logistic regression analysis of baseline characteristics and biomarkers associated with conception in women with URPL.

Variable	Crude OR (95% CI)	*p*	Adjusted OR (95%CI) †	*p*
Clinical Characteristics				
Age (per year)	0.99 (0.90-1.10)	0.881	0.96 (0.86-1.07)	0.461
BMI (per kg/m²)	1.05 (0.92-1.19)	0.474	1.06 (0.93-1.22)	0.374
ART (Yes vs No)	1.21 (0.54-2.89)	0.654	0.53 (0.17-1.68)	0.277
No. of previous losses	1.16 (0.81-1.74)	0.442	1.26 (0.50-3.14)	0.622
Biomarkers				
Testosterone	0.51 (0.29-0.86)	0.014*	0.50 (0.28-0.89)	0.019*
Prolactin	0.98 (0.95-1.02)	0.412	–	–
eGFR	1.02 (0.96-1.08)	0.519	–	–
Platelet Aggregation (ADP)	1.00 (0.96-1.05)	0.815	–	–
Platelet Aggregation (AA)	1.00 (0.98-1.01)	0.848	–	–
Fasting Glucose	0.70 (0.29-1.66)	0.422	–	–
TSH	1.01 (0.72-1.42)	0.968	–	–
Anti-PS/PT IgG	1.03 (0.94-1.15)	0.591	–	–
Anti-PS/PT IgM	1.00 (0.96-1.05)	0.822	–	–
Homocysteine	0.86 (0.71-1.03)	0.105	–	–
TPO-Ab Positive	2.20 (0.54-14.84)	0.327	–	–

OR, Odds Ratio; CI, Confidence Interval. † Multivariable model adjusted for age, BMI, number of previous losses, ART use, and variables with *p* < 0.1 in univariate analysis.

*p < 0.05.

### Multi-omics signatures associated with conception

#### Metabolomics profiling

**Figure 2:**
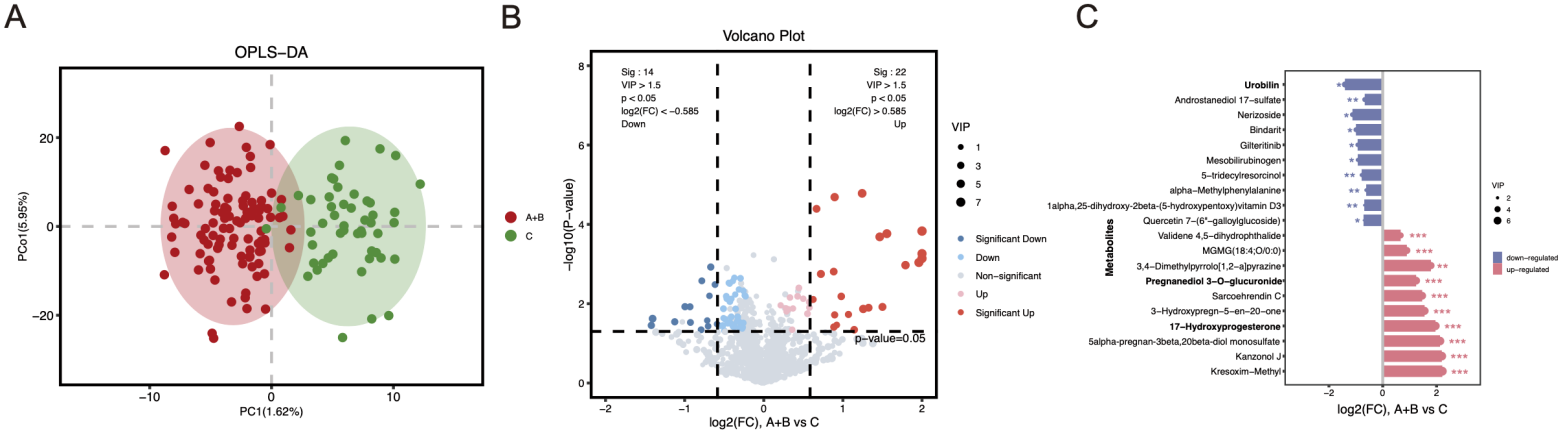
Metabolomic profiles associated with conception. **(A)** OPLS-DA score plot comparing the conception group (ongoing pregnancy + early pregnancy loss) (Group A+B, n=99, red) and the non-conception group (Group C, n=50, green). **(B)** Volcano plot of differential metabolites. Positive log2(FC) values and red dots indicate upregulation in the conception group, whereas negative log2(FC) values and blue dots indicate downregulation. Candidate metabolites were selected using VIP > 1.5, FC ≥ 1.5 or ≤ 0.67, *p* < 0.05. **(C)** Lollipop plot of top differential metabolites by log2(FC), highlighting progesterone-related metabolites and urobilin in the conception group. Asterisks indicate nominal significance levels: *p < 0.05, **p < 0.01, ***p < 0.001. OPLS-DA, orthogonal partial least squares discriminant analysis; FC, fold change; VIP, variable importance in projection.

Untargeted metabolomics identified 2110 metabolites, with instrumental stability confirmed by tight QC clustering in principal component analysis (PCA). OPLS-DA showed partial separation between the conception group (Group A + B) and the non-conception (Group C) group (**Figure 2A**). Univariate analysis identified 36 differential metabolites (VIP > 1.5, FC ≥ 1.5 or ≤ 0.67, *p* < 0.05), comprising 14 downregulated and 22 upregulated metabolites (**Figure 2B**).

Lipids and lipid-like molecules accounted for 15 of the 36 differential metabolites (**Figure 2C**). Notable upregulated metabolites in the conception group included progesterone-related compounds such as 17-Hydroxyprogesterone (FC = 3.89, *p* < 0.001), pregnanediol 3-O-glucuronide (FC = 2.37, *p* < 0.001), and pregnanetriol 3α-O-b-D-glucuronide (FC = 2.39, *p* = 0.013). Other notable metabolites included organic heterocyclic compounds and organic acids, specifically Glutamine-glutamate (FC = 2.82, *p* = 0.012), asparaginylhydroxyproline (FC = 2.51, *p* = 0.013), and the downregulated Urobilin (FC = 0.37, *p* = 0.024). Collectively, these results suggested that women who subsequently conceived had higher preconception levels of progesterone-related metabolites, which may reflect more favorable luteal-phase function.

#### Proteomics profiling

**Figure 3:**
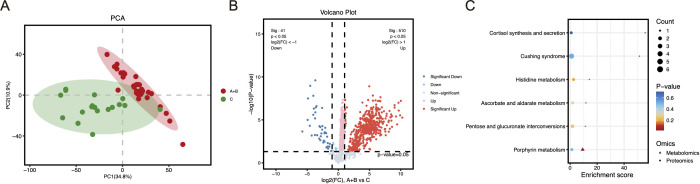
Proteomic alterations and joint pathway analysis associated with conception. **(A)** PCA plot comparing the conception group (ongoing pregnancy + early pregnancy loss) (Group A+B, n=99, red) and non-conception group (Group C, n=50, green). **(B)** Volcano plot of 551 DEPs. Positive log2(FC) values (red points) indicate upregulation in the conception group, whereas negative log2(FC) values (blue points) indicate downregulation. DEPs are defined using FC ≥ 2 or FC ≤ 0.5 and *p* < 0.05. **(C)** Joint pathway analysis integrating metabolomic and proteomic findings identifies six overlapping KEGG pathways between the conception and non-conception groups. Dot size represents the number of enriched features, and color intensity represents the pathway-level *p*-value. PCA, principal component analysis; DEP, differentially expressed protein; FC, fold change; KEGG, Kyoto Encyclopedia of Genes and Genomes.

Parallel DIA proteomics identified 2720 proteins, with a median of 7 unique peptides per protein, and 2525 proteins supported by at least 2 unique peptides. PCA showed partial separation between conception and non-conception groups (**Figure 3A**). Using filtering criteria of FC ≥ 2 or ≤ 0.5 and *nominal p* < 0.05, 551 nominally DEPs were identified, including 510 upregulated and 41 downregulated proteins in the conception group (**Figure 3B**). The majority of DEPs met q < 0.05, but given the sample size, individual protein-level findings should be interpreted cautiously.

Serpin B6 was the most significantly upregulated protein in the conception group (FC = 1224.07). Other notable upregulated proteins were encoded by SKIC3, MAP1A, ATP5MJ, SH3PXD2B, ARFGEF1, NT5C2, CYP20A1, and SCAMP1. In contrast, RAB33B, EPB41L2, CA6, GAPDHS, S100A12, IMPA1, ATF6B, KAZALD1, and GABARAPL2 were among the most downregulated (**Supplementary Table 1**). In addition, several DEPs were annotated to steroid or progesterone-related biological processes. Translocator protein, involved in C21-steroid hormone biosynthesis and response to progesterone, was markedly upregulated (FC = 31.38). Sterol carrier protein 2, annotated to progesterone biosynthetic process and sterol transport, was also upregulated (FC = 67.34). Two proteins mapped to the progesterone-mediated oocyte maturation pathway, including Guanine nucleotide-binding protein G(i) subunit alpha-1 (FC = 24.36) and Serine/threonine-protein kinase A-Raf (FC = 32.09). These findings are concordant with the metabolomic observation that progesterone-related metabolites were increased in women who subsequently conceived, but the extreme fold changes (e.g., FC > 1000) may reflect low baseline abundance rather than biologically meaningful differences. Such proteins should be prioritized for targeted validation.

### Multi-omics integration and joint pathway analysis

To further explore functional overlap between metabolic and proteomic findings, joint pathway analysis identified six overlapping KEGG pathways between the conception and non-conception groups, including cortisol synthesis and secretion, Cushing syndrome, histidine metabolism, porphyrin metabolism, ascorbate and aldarate metabolism, and pentose and glucuronate interconversions (**Figure 3C**). Among these pathways, ascorbate and aldarate metabolism and pentose and glucuronate interconversions were particularly relevant because they involved testosterone, pregnanediol 3-O-glucuronide, together with multiple DEPs described above.

### Clinical factors associated with early pregnancy loss

We evaluated clinical factors associated with early pregnancy loss using logistic regression. In univariate analysis, anti-PS/PT IgM *(p =* 0.083), prolactin *(p =* 0.041), eGFR *(p =* 0.025), ADP-induced platelet aggregation *(p =* 0.029), and arachidonic acid-induced platelet aggregation *(p =* 0.081) met the prespecified screening threshold *(p <* 0.1). After adjustment for maternal age, BMI, number of previous losses, and ART use, higher baseline prolactin was associated with higher odds of early pregnancy loss (adjusted OR 1.09, 95% CI: 1.01–1.18, *p* = 0.036). Baseline anti-PS/PT IgM showed a borderline association with the risk of early pregnancy loss (adjusted OR 1.07, 95% CI:1.00–1.14, *p* = 0.052). eGFR and platelet aggregation parameters were no longer statistically significant in adjusted analyses (**Table 3**).

**Table 3:** Multivariable logistic regression analysis of baseline characteristics and biomarkers associated with early pregnancy loss in women with URPL.

Variable		Crude OR (95% CI)	*p*	Adjusted OR (95%CI) †	*p*	Integrated OR (95%CI)	*p*
Clinical Characteristics							
Age (per year)		1.11 (0.98-1.27)	0.110	1.09 (0.91-1.31)	0.356	–	–
BMI (per kg/m²)		0.95 (0.80-1.11)	0.502	0.93 (0.77-1.11)	0.408	–	–
ART Use (Yes vs No)		1.89 (0.71-5.00)	0.198	1.79 (0.57-5.59)	0.312	–	–
No. of previous losses		0.90 (0.58-1.39)	0.623	0.78 (0.46-1.31)	0.343	–	–
Clinical biomarkers							
Anti-PS/PT IgM		1.05 (0.99-1.10)	0.083	1.07 (1.00-1.14)	0.052	1.05 (1.00-1.11)	0.068
Prolactin		1.07 (1.00-1.15)	0.041*	1.09 (1.01-1.18)	0.036*	1.07 (1.00-1.14)	0.066
eGFR		0.92 (0.86-0.99)	0.025*	0.94 (0.86-1.03)	0.179	–	–
Platelet Aggregation (ADP)		0.94 (0.89-0.99)	0.029*	0.98 (0.92-1.04)	0.471	–	–
Platelet Aggregation (AA)		0.98 (0.97-1.00)	0.081	0.99 (0.96-1.01)	0.185	–	–
Fasting Glucose		1.92 (0.70-5.22)	0.201	–	–	–	–
Anti-PS/PT IgG		1.09 (0.98-1.21)	0.128	–	–	–	–
TSH		0.92 (0.60-1.41)	0.691	–	–	–	–
Testosterone		0.53 (0.25-1.13)	0.102				
Homocysteine		1.17 (0.90-1.51)	0.237	–	–	–	–
TPO-Ab Positive		1.87 (0.49-7.19)	0.359	–	–	–	–
Metabolomic biomarkers							
Deoxycholic acid glycine conjugate	0.74 (0.61-0.91)		0.004*	0.91 (0.57-1.44)	0.688	–	–
Chenodeoxycholic acid glycine conjugate	0.66 (0.49-0.88)		0.005*	0.64 (0.25-1.59)	0.334	–	–
7α,12α-dihydroxy-3-oxocholest-4-en-27-oic acid	0.61 (0.42-0.88)		0.009*	0.56 (0.35-0.88)	0.013*	0.64 (0.44-0.95)	0.025*
Glycoursodeoxycholic acid	0.73 (0.57-0.93)		0.010*	0.72 (0.43-1.21)	0.215	–	–
SDCA-Sulfodeoxycholic acid	0.77 (0.62-0.95)		0.016*	0.72 (0.35-1.49)	0.371	–	–
Lagodeoxycholic acid	0.67 (0.49-0.92)		0.014*	1.01 (0.49-2.08)	0.982	–	–
Glycocholic acid	0.68 (0.50-0.93)		0.016*	1.40 (0.65-3.01)	0.396	–	–

The integrated model is exploratory and should not be interpreted as a validated prediction model, risk score, or clinical decision-making tool. OR, Odds Ratio; CI, Confidence Interval. † Multivariable model adjusted for age, BMI, number of previous losses, ART use, and variables with *p* < 0.1 in univariate analysis. *p < 0.05.

### Multi-omics signatures associated with early pregnancy loss

#### Metabolomics profiling

**Figure 4:**
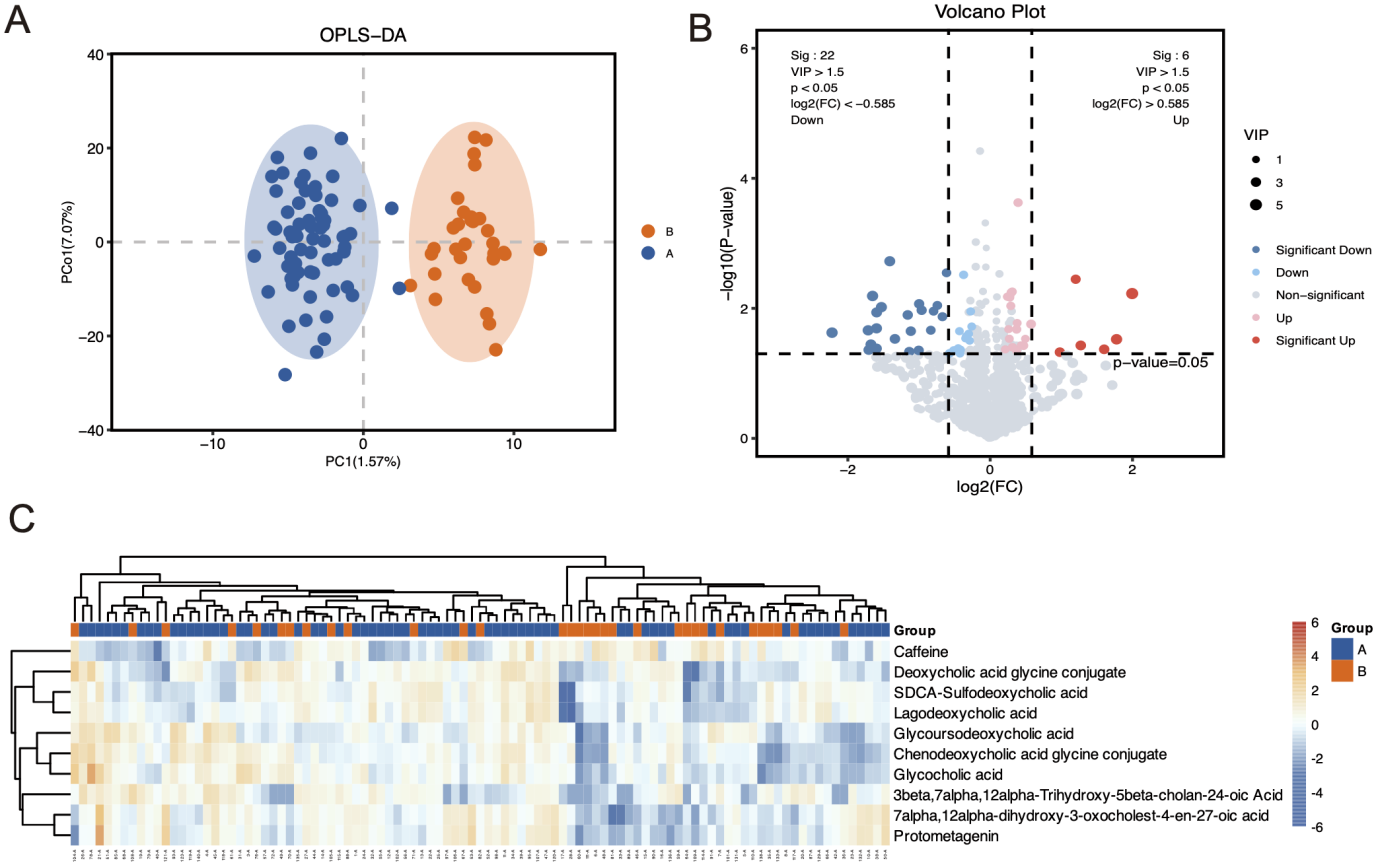
Metabolomic signatures associated with early pregnancy loss. **(A)** OPLS-DA score plot comparing ongoing pregnancy (Group A, n=67, blue) and early pregnancy loss (Group B, n=32, orange). **(B)** Volcano plot of differential metabolites between early pregnancy loss and ongoing pregnancy. Positive log2(FC) values (red dots) indicate upregulation in the early pregnancy loss group, whereas negative log2(FC) values (blue dots) indicate downregulation. Candidate metabolites were selected using VIP > 1.5, *p* < 0.05, and FC ≥ 1.5 or FC ≤ 0.67. **(C)** Hierarchical clustering heatmap of selected differential metabolites. Columns represent individual participants, rows represent metabolites, and the top annotation indicates pregnancy outcome groups. The color scale represents row-scaled metabolite abundance, with red indicating higher relative abundance and blue indicating lower relative abundance. The heatmap highlights lower bile acid-related metabolites and higher caffeine-related metabolites in the early pregnancy loss group. OPLS-DA, orthogonal partial least squares discriminant analysis; FC, fold change; VIP, variable importance in projection.

Among women who conceived, OPLS-DA indicated partial metabolic separation between ongoing early pregnancy (Group A) and early pregnancy loss (Group B) (**Figure 4A**), although the negative Q² value from permutation testing indicated limited predictive performance and supports cautious interpretation of this separation. Univariate analysis identified 28 differential metabolites (VIP > 1.5, FC ≥ 1.5 or ≤ 0.67, *p* < 0.05), including 22 down-regulated and 6 up-regulated metabolites in the early pregnancy loss group (**Figure 4B**).

The differential profile was dominated by steroids and steroid derivatives (22/28), particularly in the subclass of bile acids, alcohols, and derivatives (13/28). In the early pregnancy loss group, multiple bile acid-related metabolites were consistently lower, including deoxycholic acid glycine conjugate (FC = 0.35, *p* = 0.010), glycoursodeoxycholic acid (FC = 0.45, *p* = 0.013), chenodeoxycholic acid glycine conjugate (FC = 0.50, *p* = 0.009), glycocholic acid (FC = 0.56, *p* = 0.022), and 7α,12α-dihydroxy-3-oxocholest-4-en-27-oic acid (FC = 0.60, *p* = 0.009). In contrast, the early pregnancy loss group showed accumulation of caffeine (FC = 3.42, *p* = 0.006), 1-methylxanthine (FC = 3.04, *p* = 0.043) and 1-methyluric acid (FC = 1.97, *p* = 0.047) (**Figure 4C**, **Supplementary Figure 1**). Consistent with these findings, KEGG pathway analysis indicated enrichment in primary bile acid biosynthesis and caffeine metabolism. The caffeine-related pattern should be interpreted cautiously (18–20), because metabolite abundance may reflect both intake and individual clearance.

### Proteomic characterization of early pregnancy loss

**Figure 5:**
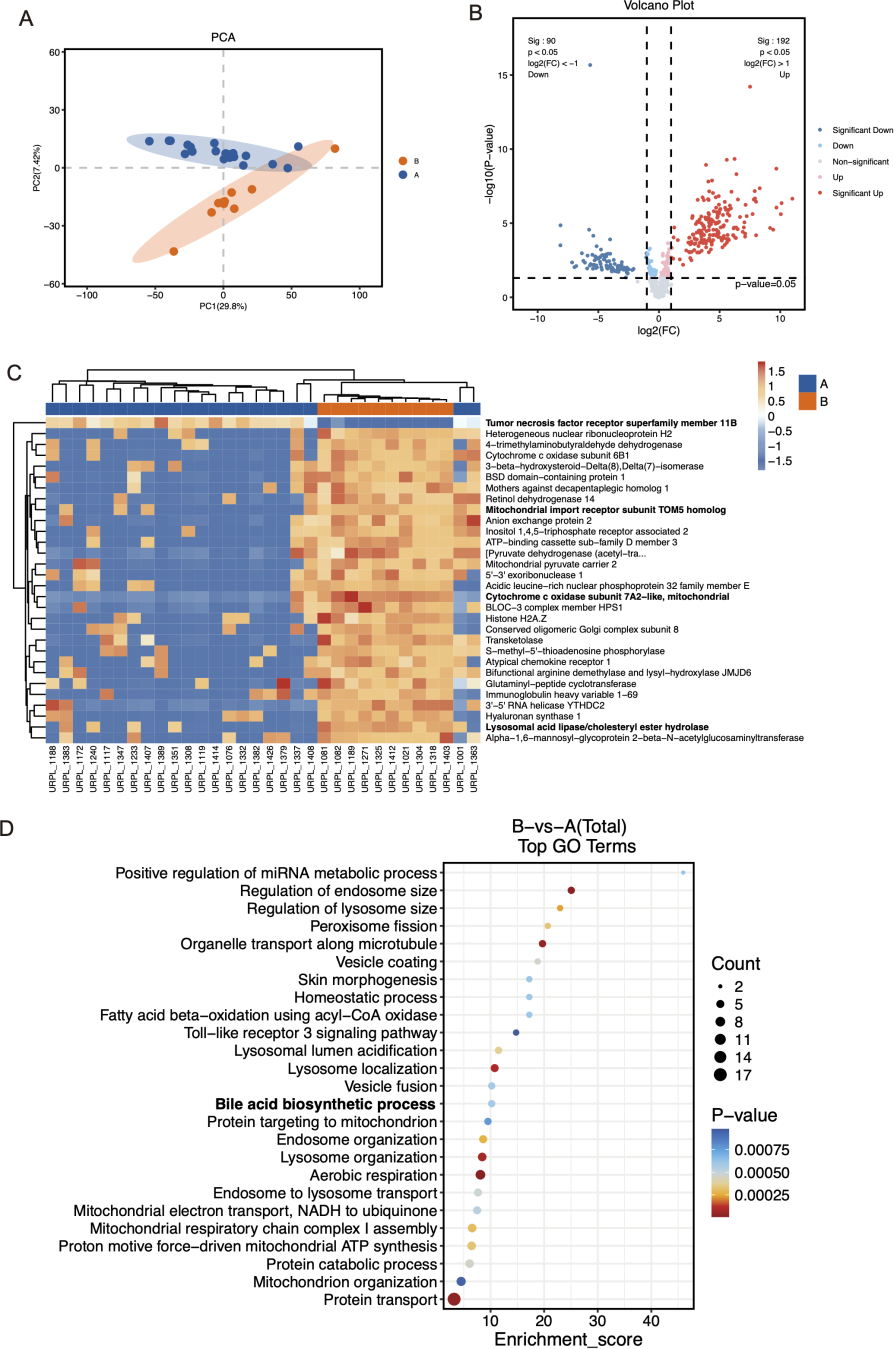
Proteomic profiles associated with early pregnancy loss. **(A)** PCA plot comparing ongoing pregnancy (Group A, n=67, blue) and early pregnancy loss (Group B, n=32, orange). **(B)** Volcano plot of 282 DEPs. Positive log2(FC) values (red dots) indicate upregulation in the early pregnancy loss group, whereas negative log2(FC) values (blue dots) indicate downregulation in the early pregnancy loss group. DEPs were defined using FC ≥ 2 or FC ≤ 0.5 and *p* < 0.05. **(C)** Hierarchical clustering heatmap of the top 30 significant DEPs in early pregnancy loss and ongoing pregnancy. Rows represent proteins and columns represent participants. Bold labels indicate representative DEPs, including tumor necrosis factor receptor superfamily member 11B, cytochrome c oxidase subunit 7A2-like, mitochondrial, lysosomal acid lipase, and mitochondrial import receptor subunit TOM5 homolog. **(D)** GO enrichment analysis of DEPs. Dot size represents the number of DEPs in each enriched term, and color intensity represents the enrichment *p*-value. The bile acid biosynthetic process was highlighted among the enriched pathways.PCA, principal component analysis; GO, Gene Ontology; FC, fold change; DEPs, differentially expressed proteins.

PCA demonstrated partial separation between the ongoing pregnancy (Group A) and early pregnancy loss groups (Group B) (**Figure 5A**). Applying the same filtering criteria (FC ≥ 2 or FC ≤ 0.5, *p* < 0.05), we identified 282 differentially expressed proteins, including 192 upregulated and 90 downregulated proteins in the early pregnancy loss group relative to the ongoing early pregnancy group (**Figure 5B**). The most prominent individual protein changes included marked downregulation of TNF receptor superfamily member 11b (FC = 0.020) and upregulation of Cytochrome c oxidase subunit 7A2-like, mitochondrial (FC = 182.59), Lysosomal Acid Lipase (FC = 820.28), and Mitochondrial import receptor subunit TOM5 homolog (FC = 1106.90) (**Figure 5C**). Importantly, GO enrichment analysis highlighted bile acid biosynthetic process among the enriched biological processes, providing proteomic support for the bile acid-related metabolomic findings in early pregnancy loss (**Figure 5D**). Several DEPs mapped to this biological process. ATP-binding cassette sub-family D member 3 (FC = 21.87) and Sterol carrier protein 2 (FC = 127.87) were also upregulated in the early pregnancy loss group, whereas Acyl-coenzyme A thioesterase 8 (FC = 0.098) and Oxysterol-binding protein 1 (FC = 0.061) were downregulated.

### Bile acid-related metabolites and exploratory clinical and metabolomic integration

**Figure 6:**
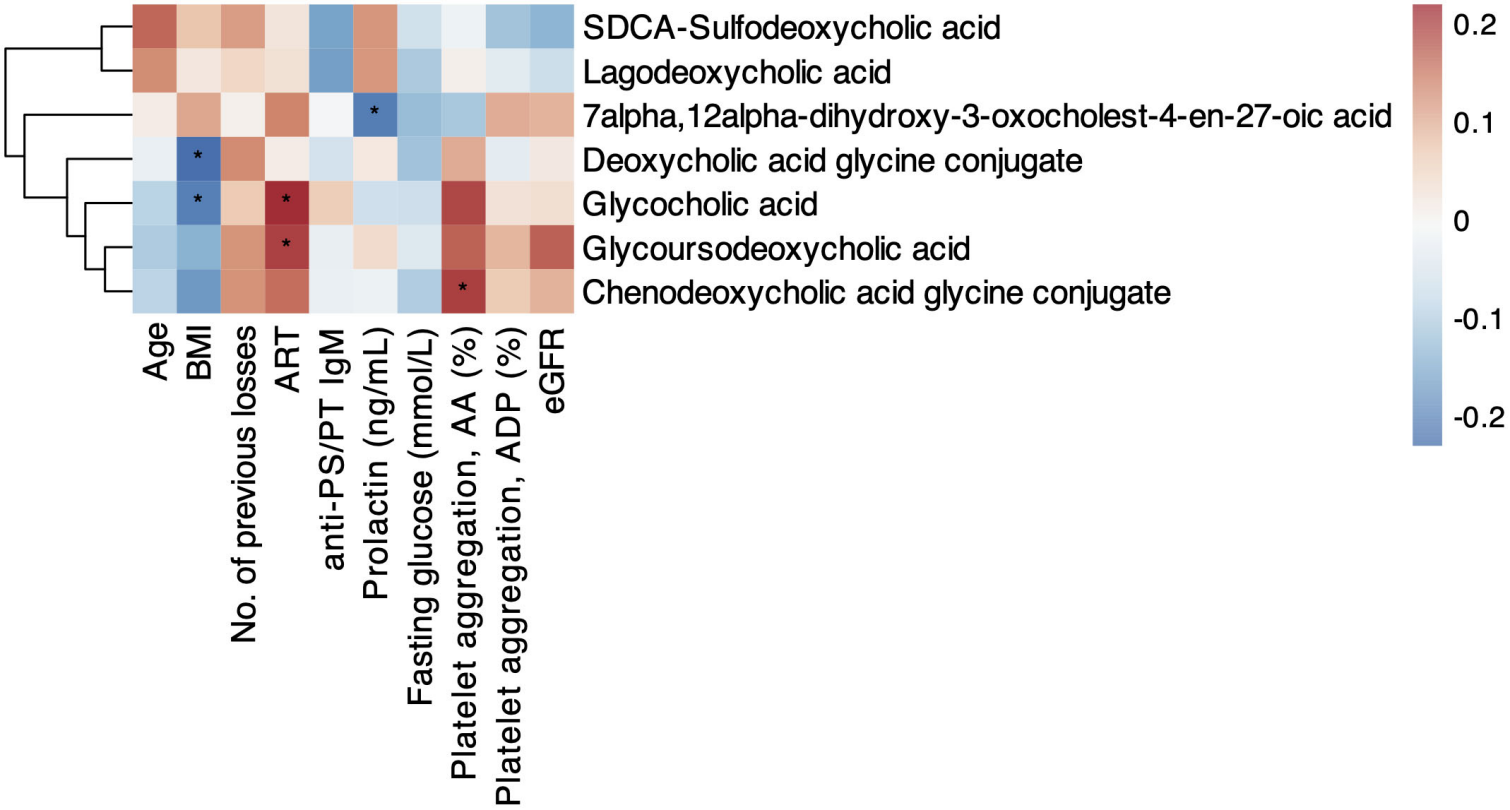
Correlation analysis of bile acid-related metabolites and clinical variables. Rows display bile acid-related metabolites identified in the early pregnancy loss analysis, and columns show clinical variables. The colors represent Pearson correlation coefficients, with red indicating positive correlations and blue indicating negative correlations. Absolute correlation coefficients remained below 0.23, indicating limited multicollinearity between the selected clinical variable and metabolic biomarkers. BMI, body mass index; ART, assisted reproductive technology; PRL, prolactin; Anti-PS/PT, anti-phosphatidylserine/prothrombin; eGFR, estimated glomerular filtration rate; AA, arachidonic acid; ADP, adenosine diphosphate; FDR, false discovery rate. * : p < 0.05.

Before integrating clinical and metabolic variables, we examined correlations between candidate metabolites and clinical indicators (**Figure 6**). All absolute correlation coefficients were below 0.23. Although six correlations were nominally significant (*p* < 0.05), including an inverse correlation between 7α,12α-dihydroxy-3-oxocholest-4-en-27-oic acid and prolactin (r = -0.212, *p* = 0.035), none remained significant after FDR adjustment. We then assessed bile acid-related metabolites. Univariate analysis identified seven bile acid-related metabolites associated with early pregnancy loss (*p* < 0.05). After adjustment for clinical confounders, higher levels of 7α,12α-dihydroxy-3-oxocholest-4-en-27-oic acid remained associated with lower odds of early pregnancy loss (adjusted OR 0.56, 95% CI: 0.35-0.88, *p* = 0.013); (**Table 3**).

In the exploratory integrated clinical and metabolomic model, 7α,12α-dihydroxy-3-oxocholest-4-en-27-oic acid remained associated with lower odds of early pregnancy loss (OR 0.64, 95% CI: 0.44–0.95, *p* = 0.025). The association of anti-PS/PT IgM (OR 1.05, 95% CI: 1.00–1.11, *p* = 0.068) and prolactin (OR 1.07, 95% CI: 1.00–1.14, *p* = 0.066) was attenuated after inclusion of this metabolite (**Table 3**).

## Discussion

In this study, we observed differences in baseline clinical and omics features associated with conception and early pregnancy loss in women with URPL. Higher preconception testosterone was associated with lower odds of conception, whereas women who conceived showed higher levels of progesterone-related metabolites. Among women who conceived, higher baseline prolactin was statistically associated with early pregnancy loss, and anti-PS/PT IgM showed a borderline association. Early pregnancy loss was also characterized by lower bile acid-related metabolites, higher caffeine and methylxanthine metabolites, with proteomic results supporting related metabolic findings. In the exploratory integrated model, higher 7α,12α-dihydroxy-3-oxocholest-4-en-27-oic acid levels were associated with a lower risk of early pregnancy loss, after accounting for selected clinical variables.

Anti-PS/PT IgM should be interpreted cautiously in this cohort. Anti-PS/PT is a non-criteria antiphospholipid antibody and is not included in the 2023 ACR/EULAR classification criteria (6). In our cohort, from which all patients with definite APS had been excluded, baseline anti-PS/PT IgM exhibited a borderline, non-significant association with early pregnancy loss. Previous studies have reported associations between anti-PS/PT antibodies and adverse pregnancy outcomes. For example, anti-PS/PT positivity is more prevalent in women with RPL than in controls, with an odds ratio of approximately 5.9 (8), consistent with prior findings by our group and others (21). Possible mechanisms include complement activation, endothelial activation, and local micro-thrombotic processes at the maternal–fetal interface (22–25), resembling the pathogenesis of APS (26). Therefore, this finding is best interpreted as a non-criteria aPL signal requiring independent validation before potential clinical use.

Prolactin provides a plausible endocrine mechanism for early pregnancy loss in this cohort. Hyperprolactinemia may suppress hypothalamic GnRH secretion and disrupt the hypothalamic-pituitary-ovarian (HPO) axis, thereby impairing follicular development, corpus luteum function, and luteal progesterone support (9, 27, 28). In a randomized bromocriptine trial of women with hyperprolactinemic recurrent miscarriage, restoration of prolactin levels was associated with a higher successful pregnancy rate (29). Experimental data also suggest that high prolactin exposure can induce granulosa-cell oxidative stress and apoptosis (30), providing a potential link between endocrine dysfunction and impaired early pregnancy support. Because URPL-specific prolactin thresholds that influence early pregnancy outcome remain undefined (4, 9, 27), the observed association should not be translated into a clinical cutoff without validation. Further studies are required to determine whether prolactin measurement, clinically meaningful thresholds, or targeted normalization possess prognostic or therapeutic value in URPL (31).

Alterations of the bile acid-related pathway were a notable finding in the early pregnancy loss group. Rather than a single isolated metabolite, the exploratory metabolomic profile showed coordinated downregulation of bile acids and correlated derivatives. In particular, downregulation of 7α,12α-dihydroxy-3-oxocholest-4-en-27-oic acid, an intermediate in bile acid synthesis, was associated with pregnancy loss. Physiological bile acid homeostasis may be relevant to the maintenance of the fetal-maternal interface (32, 33), because bile acids can function as signaling molecules through receptors such as FXR and TGR5 (34). Activation of these receptors has been shown to suppress pro-inflammatory cytokines and promote regulatory T-cell differentiation, thereby supporting immune tolerance at the maternal-fetal interface (35). Because the gut microbiota plays a pivotal role in shaping the systemic bile acid pool through biotransformation, it may represent an upstream regulator of this process. Recent work has linked perturbations in gut microbiota-derived bile acids to dysregulated circulating immune cell subsets in URPL (36). Clinically, these findings suggest that bile acid metabolites merit validation as prognostic markers in larger cohorts and provide a basis for further investigation of bile acid and microbiota-related metabolism in URPL.

Another noteworthy finding was the higher abundance of caffeine and downstream methylxanthine metabolites in the early pregnancy loss group. Prior meta-analyses have reported increased risk of pregnancy loss with higher caffeine intake, noting an estimated 14%–19% increase per 100–150 mg/day increment (19, 37). Accordingly, professional guidelines commonly recommend limiting caffeine intake during pregnancy, often to approximately 200 mg/day (18, 38). Our findings are consistent with this broader literature but caffeine and methylxanthine abundances reflect both intake and clearance. Mechanistically, caffeine is primarily metabolized by cytochrome P450 1A2 (CYP1A2) enzyme, and individual variation in CYP1A2 activity may prolong the half-life of caffeine and intensify fetal exposure (20, 39). Because dietary intake and caffeine consumption were not recorded in detail, caffeine and methylxanthine abundances should be interpreted as exploratory.

The association between higher preconception testosterone and lower odds of conception is biologically plausible. Elevated androgens may impair folliculogenesis, ovulatory competence, and endometrial receptivity, mechanisms that overlap with PCOS-related reproductive dysfunction (40, 41). PCOS was not formally diagnosed, so the contribution of unmeasured PCOS-related features to the testosterone-conception association cannot be excluded (42). Conversely, women who conceived showed higher levels of progesterone-related metabolites, which may reflect more favorable luteal function and an endocrine milieu supportive of implantation. Although studies such as the PROMISE trial have highlighted heterogeneity in the benefit of progesterone after conception (43), our findings support further investigation of whether progesterone-related metabolic status and individualized luteal support before or around conception may influence reproductive outcomes in URPL.

A conceptual strength of this study is the distinction between factors associated with conception and factors associated with early pregnancy loss. Many RPL studies focus exclusively on post-implantation recurrence, but failure to conceive is also clinically important. The prospective cohort design reduces the risk of confounding, and the combination of clinical biomarkers with metabolomic and proteomic profiling allowed multiple features to be evaluated within the same cohort.

Certain limitations must be acknowledged. First, although the cohort was prospective, the number of early pregnancy losses was modest relative to the dimensionality of untargeted metabolomics and DIA proteomics, thereby increasing the risk of false-positive findings and model over-fitting. Second, baseline metabolite and protein levels are influenced by diet, circadian timing, menstrual cycle phase, microbiome-related exposures, and other unmeasured factors. The absence of detailed dietary, caffeine intake, sampling-time, menstrual-phase, and microbiome data limits causal interpretation of the bile acid and caffeine findings. Third, while many DEPs met FDR-adjusted significance thresholds, the number of DEPs was large relative to the sample size. Proteomic findings should therefore be interpreted primarily at the pathway enrichment level rather than as individually validated protein associations. Fourth, clinical variables were screened at a nominal threshold of *p* < 0.1 for inclusion in multivariable models, without formal correction for multiple clinical comparisons, which may inflate type I error. In addition, the clinical, metabolic and proteomic associations reported here should be interpreted cautiously and require validation in independent cohorts. Finally, treatment during follow-up was not randomized and may have influenced reproductive outcomes in ways that could not be fully accounted for.

In conclusion, elevated testosterone levels were associated with reduced odds of conception among women with URPL. Among those who conceived, early pregnancy loss was associated with higher prolactin, borderline elevated anti-PS/PT IgM, lower bile acid-related metabolites, and higher caffeine and methylxanthine metabolites. 7α,12α-dihydroxy-3-oxocholest-4-en-27-oic acid was associated with lower odds of early pregnancy loss. These exploratory findings are preliminary, and require validation in external cohorts.

## Data Availability

The raw untargeted metabolomics and data-independent acquisition (DIA) proteomics data generated and analyzed in this study have been deposited in the OMIX repository, China National Center for Bioinformation (https://ngdc.cncb.ac.cn/omix), and are publicly accessible. The metabolomics data are available under accession number OMIX017150 (https://ngdc.cncb.ac.cn/omix/release/OMIX017150), and the proteomics data are available under accession number OMIX017149 (https://ngdc.cncb.ac.cn/omix/release/OMIX017149). Any additional information required to reanalyze the data reported in this paper is available from the corresponding author upon reasonable request. No custom algorithms or software were developed for this study.
