# Supplementary material for: Clinical, metabolomic, and proteomic profiles associated with reproductive outcomes in unexplained recurrent pregnancy loss
Source: Front Endocrinol (Lausanne). 2026 Jul 10;17:1875194. doi: 10.3389/fendo.2026.1875194 (PMC13395727; doi:10.3389/fendo.2026.1875194)
Supplement: Supplementary file 1 [file DataSheet1.docx]

| Accession | Gene Name | Description | FC | log2FC | p-value | q-value | Regulation |
| --- | --- | --- | --- | --- | --- | --- | --- |
| P35237 | SERPINB6 | Serpin B6 | 1224.07 | 10.26 | 5.54E-06 | 1.07E-04 | Up |
| Q6PGP7 | SKIC3 | Superkiller complex protein 3 | 1039.52 | 10.02 | 6.16E-08 | 1.11E-05 | Up |
| P78559 | MAP1A | Microtubule-associated protein 1A | 960.35 | 9.91 | 3.01E-06 | 7.96E-05 | Up |
| P56378 | ATP5MJ | ATP synthase subunit ATP5MJ, mitochondrial | 822.41 | 9.68 | 8.43E-07 | 4.51E-05 | Up |
| A1X283 | SH3PXD2B | SH3 and PX domain-containing protein 2B | 800.43 | 9.64 | 1.02E-07 | 1.39E-05 | Up |
| Q9Y6D6 | ARFGEF1 | Brefeldin A-inhibited guanine nucleotide-exchange protein 1 | 690.14 | 9.43 | 3.42E-05 | 2.73E-04 | Up |
| P49902 | NT5C2 | Cytosolic purine 5'-nucleotidase | 520.32 | 9.02 | 3.68E-07 | 3.13E-05 | Up |
| Q6UW02 | CYP20A1 | Cytochrome P450 20A1 | 518.87 | 9.02 | 1.95E-07 | 2.31E-05 | Up |
| O15126 | SCAMP1 | Secretory carrier-associated membrane protein 1 | 389.98 | 8.61 | 6.54E-05 | 3.80E-04 | Up |
| P60520 | GABARAPL2 | Gamma-aminobutyric acid receptor-associated protein-like 2 | 0.074 | -3.76 | 5.85E-04 | 1.54E-03 | Down |
| Q96I82 | KAZALD1 | Kazal-type serine protease inhibitor domain-containing protein 1 | 0.066 | -3.93 | 4.92E-07 | 3.43E-05 | Down |
| Q99941 | ATF6B | Cyclic AMP-dependent transcription factor ATF-6 beta | 0.062 | -4.01 | 1.59E-08 | 8.64E-06 | Down |
| P29218 | IMPA1 | Inositol monophosphatase 1 | 0.062 | -4.02 | 2.56E-06 | 7.67E-05 | Down |
| P80511 | S100A12 | Protein S100-A12 | 0.052 | -4.27 | 6.70E-04 | 1.71E-03 | Down |
| O14556 | GAPDHS | Glyceraldehyde-3-phosphate dehydrogenase, testis-specific | 0.044 | -4.52 | 1.11E-09 | 1.14E-06 | Down |
| P23280 | CA6 | Carbonic anhydrase 6 | 0.034 | -4.88 | 3.27E-08 | 1.11E-05 | Down |
| O43491 | EPB41L2 | Band 4.1-like protein 2 | 0.033 | -4.93 | 1.02E-05 | 1.44E-04 | Down |
| Q9H082 | RAB33B | Ras-related protein Rab-33B | 0.018 | -5.77 | 2.27E-04 | 8.01E-04 | Down |

Supplementary Table 1. Prominent differential proteins identified in the study

**Supplementary Figure 1. Boxplot of representative differential metabolites**
